# A Low-Diversity Microbiota Inhabits Extreme Terrestrial Basaltic Terrains and Their Fumaroles: Implications for the Exploration of Mars

**DOI:** 10.1089/ast.2018.1870

**Published:** 2019-03-06

**Authors:** Charles S. Cockell, Jesse P. Harrison, Adam H. Stevens, Samuel J. Payler, Scott S. Hughes, Shannon E. Kobs Nawotniak, Allyson L. Brady, R.C. Elphic, Christopher W. Haberle, Alexander Sehlke, Kara H. Beaton, Andrew F.J. Abercromby, Petra Schwendner, Jennifer Wadsworth, Hanna Landenmark, Rosie Cane, Andrew W. Dickinson, Natasha Nicholson, Liam Perera, Darlene S.S. Lim

**Affiliations:** ^1^ UK Centre for Astrobiology, School of Physics and Astronomy, University of Edinburgh, Edinburgh, UK.; ^2^ Aquatic Biogeochemistry Research Unit, Ecosystems and Environment Research Programme, Faculty of Biological and Environmental Sciences, University of Helsinki, Helsinki, Finland.; ^3^ Turku Centre for Biotechnology, University of Turku and Åbo Akademi University, Turku, Finland.; ^4^ Department of Geosciences, Idaho State University, Pocatello, Idaho, USA.; ^5^ School of Geography and Earth Sciences, McMaster University, Hamilton, Canada.; ^6^ NASA Ames Research Center, Mountain View, California, USA.; ^7^ Mars Space Flight Facility, Arizona State University, Tempe, Arizona, USA.; ^8^ KBRwyle, NASA Johnson Space Center, Houston, Texas, USA.; ^9^ Biomedical Research & Environmental Sciences Division (SK), NASA Johnson Space Center, Houston, Texas, USA.; ^10^ Bay Area Environmental Research Institute (BAERI), Moffett Field, California, USA.

**Keywords:** Mars, Life, Basalts, Bacteria, Weathering, Human exploration

## Abstract

A major objective in the exploration of Mars is to test the hypothesis that the planet hosted life. Even in the absence of life, the mapping of habitable and uninhabitable environments is an essential task in developing a complete understanding of the geological and aqueous history of Mars and, as a consequence, understanding what factors caused Earth to take a different trajectory of biological potential. We carried out the aseptic collection of samples and comparison of the bacterial and archaeal communities associated with basaltic fumaroles and rocks of varying weathering states in Hawai‘i to test four hypotheses concerning the diversity of life in these environments. Using high-throughput sequencing, we found that all these materials are inhabited by a low-diversity biota. Multivariate analyses of bacterial community data showed a clear separation between sites that have active fumaroles and other sites that comprised relict fumaroles, unaltered, and syn-emplacement basalts. Contrary to our hypothesis that high water flow environments, such as fumaroles with active mineral leaching, would be sites of high biological diversity, alpha diversity was lower in active fumaroles compared to relict or nonfumarolic sites, potentially due to high-temperature constraints on microbial diversity in fumarolic sites. A comparison of these data with communities inhabiting unaltered and weathered basaltic rocks in Idaho suggests that bacterial taxon composition of basaltic materials varies between sites, although the archaeal communities were similar in Hawai‘i and Idaho. The taxa present in both sites suggest that most of them obtain organic carbon compounds from the atmosphere and from phototrophs and that some of them, including archaeal taxa, cycle fixed nitrogen. The low diversity shows that, on Earth, extreme basaltic terrains are environments on the edge of sustaining life with implications for the biological potential of similar environments on Mars and their exploration by robots and humans.

## 1. Introduction

The planet Mars, which sits in the outer regions of the so-called “habitable zone,” is today a cold desert-like world dominated by basaltic lithologies and their alteration products (Wyatt *et al.,* 2004). The abundant evidence for a hydrologically more active past (*e.g.,* Carr, 1986; McKay and Davis, 1991; Cabrol and Grin, 2001; Haberle *et al.,* 2001; Head *et al.,* 2003; Gendrin *et al.,* 2005; Grotzinger *et al.,* 2005; Poulet *et al.,* 2005; Bibring *et al.,* 2006; Ehlmann *et al.,* 2009, 2011; Smith *et al.,* 2009; Grindrod *et al.,* 2012; Lasue *et al.,* 2013; Martínez and Rennó, 2013), particularly in the Noachian (circa 4.1–3.7 Ga) but extending episodically even to the present-day, raises the question of whether that planet hosted habitable conditions and whether those habitable environments hosted life.

Investigations of Gale Crater by the Mars Science Laboratory (MSL) suggest that habitable environments that contained potentially energy-yielding redox couples existed on Mars (Grotzinger *et al.,* 2013). Habitable conditions have also been suggested for the Phoenix landing site in the north polar region of Mars (Stoker *et al.,* 2010). Evidence for lacustrine environments in Gale Crater, with redox gradients and potentially diverse valence states of iron and sulfur (Hurowitz *et al.,* 2017), further suggest the presence of conditions that were conducive to life.

To determine whether these environments could have hosted life and what biomass was sustainable, one approach is to investigate the biomass and diversity of life in basaltic terrains on Earth that are exposed to transient meteoric and magmatic aqueous alteration. On Earth, a vast quantity of organic carbon is fixed by photosynthesis, and it provides carbon and electron donors for life. Another waste product of photosynthesis, oxygen, which is in high abundance in the near-surface environment, acts as an electron acceptor in energy production by life, a gas only found at low (∼0.15%) concentrations in the martian atmosphere. Thus, even the most extreme basaltic terrains on Earth should be regarded as highly optimistic end-members of microbial biomass and diversity that such environments can sustain. Nevertheless, investigations of microbial communities within these materials provide us with a basis to understand how life on our own planet interacts with such materials and thus to make reasoned inferences about the biological potential of these materials elsewhere.

Even if we eventually find that large areas of Mars were, or are, uninhabitable, studying the biomass and diversity of life in Earth's extreme basaltic terrains provides us with information required to determine what additional environmental, physical, and chemical conditions pushed martian environments over the edge into becoming uninhabitable. Thus, an interest in the microbiology of extreme Mars analog environments on Earth is not predicated on assumptions about the presence and extent of life on Mars but rather by an interest in establishing an empirical foundation from which to assess the biological potential of that planet and the methods we need to explore it effectively.

This work can also inform our understanding of Earth. Given the low oxygen and presumably lower organic carbon availability on basaltic landmasses on Archean Earth (Rudnick, 1995; Smithies *et al.,* 2005; Nisbet *et al.,* 2007), martian basalts may provide an analog to the early habitability of our planet. By investigating and comparing the biological potential of basalts on present-day Earth and eventually martian basaltic terrains, we will be able to construct a much clearer picture of how our own planet transitioned from life in early anoxic basaltic terrains to its current biological state. Martian basalts and their weathering products will eventually provide either an early Archean biological analog or an Archean-like abiotic control in these investigations.

The BASALT (Biologic Analog Science Associated with Lava Terrains) research program is a 4-year investigation of the geology, geochemistry, and biology of selected Mars-like basaltic terrains funded by the NASA Planetary Science and Technology through Analog Research (PSTAR) program in which sample acquisition was carried out under simulated extravehicular activity (EVA) constraints and strict aseptic control (Lim *et al.,* 2019). Therefore, the acquisition of the samples described in this paper provided the basis for optimizing EVA protocols for human explorers (Beaton *et al.,* 2019; Brady *et al.,* 2019; Payler *et al.,* 2019; Stevens *et al.,* 2019).

In this study, we characterize the biomass and diversity of life in basaltic features. Our work focused on fumaroles that ranged from relict to active and intermediate states. We compared the biota within these materials to that in unaltered and meteorically altered basalt. Our work sought to address four hypotheses relevant to life in basaltic terrains (Section 2.2). We primarily describe our results from Hawai‘i Volcanoes National Park from where it was possible to obtain the most comprehensive set of microbial community data, but we make qualitative comparisons with data obtained in the Craters of the Moon National Monument and Preserve in Idaho. Our data show the low diversity and biomass associated with extreme fumarolic volcanic environments, and we discuss the implications for the biological potential of similar environments on Mars and their exploration.

## 2. Methods

### 2.1. Field sites

**FIG. 1.**
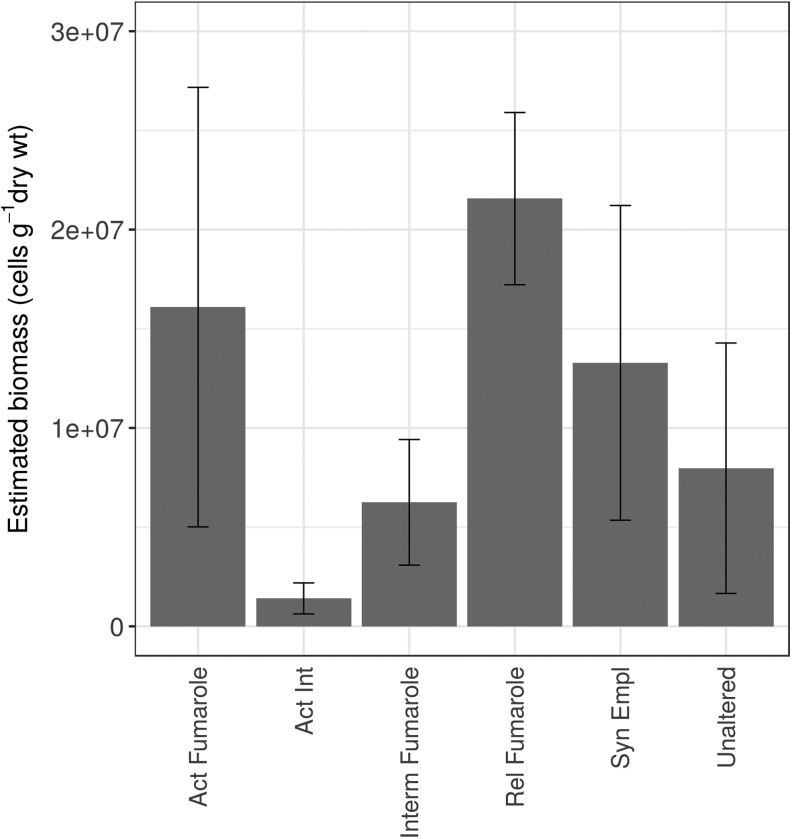
Biomass estimates for each material type for the four samples used for diversity analysis. The data are shown as means ± standard error.

The work described in this paper primarily focuses on the microbiota associated with basaltic terrains within Hawai‘i Volcanoes National Park (Lim *et al.,* Fig. 1, 2019). These rocks are formed in volcanic eruptions associated with the movement of the Pacific tectonic plate over the mantle-derived Hawaiian hot spot. The samples examined in this study were obtained in the Mauna Ulu region, a volcanic cone associated with the Kilauea volcano that is currently in the shield stage of development. Over 90% of the volcano hosts lavas less than 1500 years old, although those of Mauna Ulu are much more recent (Tilling *et al.,* 2010). The fumaroles on the volcano are primarily nonsulfurous, and weathering is mostly driven by circulation of meteoric water.

Although the martian crust is enriched in iron compared to the bulk composition of Earth (McSween *et al.,* 2009), the martian surface is dominated by basaltic materials. The basaltic materials of Hawai‘i and their alteration products have been recognized to provide useful geological and geochemical analogies to martian surface processes, particularly in early epochs when water-rock interactions were more widespread (Hughes *et al.,* 2019).

We also collected samples in the Great Rift–Craters of the Moon system in Idaho, which lies within the eastern Snake River Plain geologic province. Contrary to most basalts on the eastern Snake River Plain, these lava flows are chemically evolved from their parent basalts with possible inclusion of components from country rocks during ascent and emplacement. They were emplaced about 2–15 thousand years ago along the Great Rift, with many of the lavas younger than ∼2.4 ka. Their morphologies and geochemical compositions, including rocks with enriched silica compositions, provide a suitable geological analog for similar types of volcanism and basaltic products found on Mars. Samples were acquired from the Big Craters and Highway Flows, two of the youngest units in the Craters of the Moon lava field, as both have minimal human traffic.

A thorough description of the geological context of our field sites and their analogies to Mars can be found in Hughes *et al.* (2019).

### 2.2. Study hypotheses

In this study, our work focused on the microbiota associated with sites of past and present fumarolic activity. Fumaroles are sites of water-rock interactions and therefore provide potentially suitable places for life because of the presence of water flow, chemical disequilibria, and diverse minerals and leached elements. In the case of our field sites, the meteorically altered fumaroles can be considered analogous to volcanically active regions on past and even recent Mars where groundwater is entrained in local hydrothermal systems. Even after the cessation of active fumarolic activity, the weathered minerals that remain might be anticipated to provide a geochemically enriched environment for life compared to unaltered basaltic rocks.

We investigated four fumarolic environments from active to relic fumaroles and two stages in between. To provide a control comparison, we also investigated unaltered basaltic rock and rock altered by high-temperature aqueous or gaseous processes during emplacement (syn-emplacement material).

Our study was underpinned by four hypotheses. They were as follows:

(1) Basaltic rocks and their associated fumaroles, on account of their elementally diverse mineral composition, high concentrations of iron, and the presence of other biologically useful elements such as copper and magnesium, will host a high diversity and biomass of life.
(2) Fumaroles, on account of aqueous activity and diverse mineral suites formed through weathering, will be sites of enhanced microbial diversity and biomass compared to less altered rocks.
(3) As fumarolic activity declines, on account of less sustained aqueous activity, both microbial diversity and biomass will also decline.
(4) Unaltered basaltic rocks, on account of their relatively lower mineral diversity and lack of porous, permeable secondary minerals compared to fumaroles, will harbor a comparatively lower diversity and biomass of microbial life.

### 2.3. Sample collection

Samples were collected during simulated martian EVA as described by Lim *et al.* (2019), Beaton *et al.* (2019), Brady *et al.* (2019), Payler *et al.* (2019), and Stevens *et al.* (2019). Triplicate samples were collected from two separate examples of each alteration state under aseptic conditions (Supplementary Table S1; Supplementary Data are available at https://www.liebertpub.com/suppl/doi/10.1089/ast.2018.1870). Samples were typically ∼50–150 g in size (hand-held size). Where a rock hammer was used to loosen or break samples, the hammer was flame sterilized. Samples were collected into sterile Whirl-Pak bags and double bagged. For DNA analysis, samples were immediately frozen in a domestic freezer and shipped in a frozen condition in dry ice (-78.5°C) before processing in the laboratory. For cell enumerations, samples were refrigerated at 4°C before processing in the laboratory. However, on account of difficulties in cell enumeration, our cell enumeration data were ultimately estimated from DNA quantification (see below).

In Hawai‘i, material from the following sample types were gathered: (1) *Active fumarole.* This was material exposed to active venting within a fumarole. The material was collected where it was directly exposed to steam venting, at temperatures of above 70°C, measured by handheld forward-looking infrared (FLIR) thermometer. (2) *Active intermediate.* This was material from the periphery of one of the active fumaroles sampled in (1), but not exposed directly to active venting. (3) *Intermediate fumarole.* This was material associated with fumaroles showing low levels of activity and/or transient activity, with temperatures between 30°C and 70°C. (4) *Relict fumarole.* This was material associated with an inactive fumarole, with evidence of fumarolic alteration but temperature close to ambient. (5) *Unaltered*. This was material associated with unweathered basalt. (6) *Syn-emplacement.* This was material associated with basalt altered during its emplacement primarily by magmatically associated water and gas or meteoric water associated with the heated environment around the lava. Alteration is associated with oxidation of the material.

We also collected samples in the Craters of the Moon system in Idaho. The sample set was not as extensive as the Hawai‘i set and was not focused on as wide a range of material types. The samples were collected, stored, processed, and phylogenetically examined in an identical way to the Hawai‘i samples. Six sets of samples were collected from unaltered basalt (*Unaltered*), syn-emplacement material (*Syn Empl*), and cold altered material (*Cold*). The latter material was associated with post-eruption meteoric water weathering. In this paper, we use these data to make qualitative comparisons with the microbiota in Hawai‘i, but we test our principal hypotheses using the Hawai‘i data set.

A detailed geochemical and geological discussion of these different materials and their parent rock can be found in Hughes *et al.* (2019).

### 2.4. DNA extraction

Samples were thawed and crushed on sterile aluminum foil in a sterile flow hood using a flame-sterilized hammer until a powder had been created. We crushed the samples to determine the whole rock community composition. The powder was homogenized using a sterile ceramic pestle and mortar. Powder from each sample was separated into three 10 g aliquots, which were used to extract DNA. DNA extractions were performed using the PowerMax DNA extraction kit (MO BIO Laboratories, Carlsbad, CA, USA) according to the manufacturer's instructions.

### 2.5. DNA and biomass quantification

After extraction, the concentration of DNA was quantified using a Qubit 4 fluorometer using the dsDNA High Sensitivity Assay Kit (ThermoFisher Scientific, Waltham, MA, USA) according to the manufacturer's instructions (Supplementary Table S3). While direct cell enumeration was attempted using SYBR Green and Gold dyes (ThermoFisher Scientific, Waltham, MA, USA) on gently crushed rock material, this was prevented by background fluorescence and interference from minerals. Therefore, cell abundances were estimated using DNA concentration data under the assumption that a cell contains 8 fg of DNA as an upper limit (*e.g.,* Torsvik and Goksoyr, 1978). While this approach is subject to several uncertainties, it can nevertheless be used to obtain a meaningful between-sample comparison of the biomass sustained by the different material types examined in our study.

### 2.6. 16S rRNA gene sequencing and sequence processing

DNA extracted from the samples was sent for Illumina MiSeq sequencing at Research and Testing (Texas, USA). Bacterial sequencing was carried out with primers 28F (5′-GAGTTTGATCNTGGCTCAG-3′) and 338R (5′-GCTGCCTCCCGTAGGAGT-3′). Archaeal sequencing was carried out with primers 517F (5′-GCYTAAAGSRNCCGTAGC-3′) and 909R (5′-TTTCAGYCTTGCGRCCGTAC-3′).

Raw sequence data were processed using micca v. 1.6.2 (http://micca.org) (Albanese *et al.,* 2015). Paired-end reads were merged using the command “micca mergepairs” (Supplementary Table S2; Rognes *et al.,* 2016), and primer sequences were trimmed using “micca trim” (Martin, 2011). Quality filtering was performed using “micca filter” (see Supplementary Table S2; Rognes *et al.,* 2016). The command “micca otu” was used for chimera filtering and operational taxonomic unit (OTU) clustering, with the clustering step employing a *de novo* greedy clustering algorithm with a 97% similarity threshold (parameters -d 0.97 -c) (Rognes *et al.,* 2016). Taxonomic assignments were carried out with “micca classify” and RDP Classifier version 2.11 (Wang *et al.,* 2007). Sequence lengths (Supplementary Table S2) were as follows: bacterial sequences: Hawai‘i (277), Idaho (275); archaeal sequences: Hawai‘i (308), Idaho (308).

Further data processing was carried out using R v. 3.4.3 (R Core Team, 2017) and the “phyloseq” package (McMurdie and Holmes, 2013). To improve between-sample comparability, samples with low sequencing depth were omitted, based on the visual inspection of rarefaction curves. Further samples were randomly discarded from the Hawai‘i bacterial data set to obtain a balanced design for statistical analysis, with four biological replicates retained for each sample type (see Supplementary Table S3 for DNA concentrations and Supplementary Fig. S2 for rarefaction curves showing samples that were used for downstream analyses). This version of the data set was used for analyses of alpha diversity (details in Section 2.7). For other statistical analyses, phyla that occurred only once within a data set (this step resulted in the removal of a single archaeal phylum Diapherotrites) or were annotated as “NA” were filtered from the data set as outlined in Callahan *et al.* (2016). Chloroplast sequences (based on RDP Classifier and BLASTn results; http://blast.ncbi.nlm.nih.gov) were removed from both the Hawai‘i and Idaho bacterial data sets. For the Hawai‘i bacterial data set, a further denoising step was implemented using a 5% prevalence threshold (see Callahan *et al.,* 2016).

We were not able to obtain amplifiable DNA from negative controls (extractions run without sample introduced into the DNA kit). We were able to obtain amplifiable DNA in kit eluent from negative controls when the solution was concentrated five times through a DNA binding column. In this case, none of the taxa matched those that we obtained from the samples and dominant taxa affiliated with Corynebacteriaceae, Staphylococcaceae (data not shown). The implementation of a 5% prevalence step further ensures that the taxa we present are rock-derived organisms.

In this paper, we examined taxa at the phylum and class levels since this was sufficient to address our hypotheses and assignments at these levels are usually more reliable than at the genus or species level. However, when discussing specific samples and functional capabilities we report the assigned genera where appropriate.

Bacterial and archaeal 16S rRNA gene sequences supporting the results of this article are available in the GenBank Database (http://www.ncbi.nlm.nih.gov/genbank) under accession numbers MH102421–MH104609 (Hawai‘i bacteria), MH102015–MH102034 (Hawai‘i archaea), project accession number SAMN09430385 (Idaho bacteria), and MH102035–MH102053 (Idaho archaea).

### 2.7. Statistical analysis

#### 2.7.1. Alpha diversity

Observed OTU frequencies, Chao1 richness indices (Chao, 1984), and Shannon's diversity (*H’*) indices (Shannon, 1948) were calculated for the Hawai‘i bacterial data set using the plot_richness() function of the R package “phyloseq” (McMurdie and Holmes, 2013). During this step, singletons and doubletons were retained. Subsequently, a 5% prevalence filter was used to remove low-prevalence OTUs. Following visual inspection of diagnostic plots, Shannon's diversity values were compared using the function aov() to conduct a one-way analysis of variance (ANOVA) with “alteration type” as the factor, followed by a *post hoc* Tukey's Honest Significant Difference (HSD) test using the function TukeyHSD().

We did not rarefy all samples to the same read number, as there is increasing evidence for drawbacks to this process (*e.g.,* McMurdie and Holmes, 2014). Nevertheless, we compared nonrarefied and rarefied data sets to validate our approach. Alpha diversity estimates between rarefied and nonrarefied data sets were highly similar, as were the observed differences between samples. Comparing multivariate ordinations of nonrarefied versus rarefied data also showed comparable patterns of sample clustering between both data sets. Based on these results, we are confident that, in the case of our data, using nonrarefied sequences allowed for reliable between-sample comparisons without a major loss of information that often occurs when rarefying sequences to an equal sampling depth.

#### 2.7.2. Multivariate analyses

A Bray-Curtis resemblance matrix calculated from untransformed relative abundance values of OTUs (Hawai‘i bacterial data) was used to derive a nonmetric multidimensional scaling (nMDS) ordination using “phyloseq” (McMurdie and Holmes, 2013). Comparing nMDS ordinations based on untransformed versus Hellinger-transformed relative abundance values indicated that data transforming had no major influence on sample clustering (data not shown). Comparing ordinations for data sets where singletons and doubletons were included or removed prior to imposing the 5% prevalence filter also showed similar patterns of sample clustering. The Bray-Curtis matrix was used to conduct a one-way permutational analysis of variance (PERMANOVA) with “alteration type” as the factor, using the adonis() function of the package “vegan” (999 permutations; Oksanen *et al.,* 2018). *Post hoc* pairwise comparisons (999 permutations) were performed using the pairwise.perm.manova() function of the package “RVAideMemoire” (Hervé, 2018), including a correction for multiple testing using the Benjamini-Hochberg method (Benjamini and Hochberg, 1995). To discern whether differences between samples were due to location and/or dispersion effects, a test for the homogeneity of multivariate dispersions (PERMDISP; Anderson, 2006) was conducted using the functions betadisper() and permutest() in “vegan” (999 permutations; Oksanen *et al.,* 2018). PERMDISP is often used as an accompaniment to PERMANOVA and enables between-sample comparisons of group member distances to the sample centroid (Anderson, 2006).

## 3. Results

### 3.1. Biomass quantification

An estimated biomass (mean ± standard error as % of mean) based on DNA extraction values was obtained for the different materials collected in Hawai‘i (Fig. 1; Supplementary Table S3) by assuming that there is 8 fg of DNA per cell in each of the material types. The estimated biomass (cells g^−1^) in order from highest to lowest mean was as follows: Rel Fumarole (2.16 × 10^7^ ± 10.04); Act fumarole (1.61 × 10^7^ ± 97.11); Syn Empl (1.32 × 10^7^ ± 79.18); Unaltered (7.97 × 10^6^ ± 6.91); Int Fumarole (6.25 × 10^6^ ± 63.60); Act Int (1.40 × 10^6^ ± 74.07). The results are shown in Fig. 1.

### 3.2. Alpha diversity

**FIG. 2.**
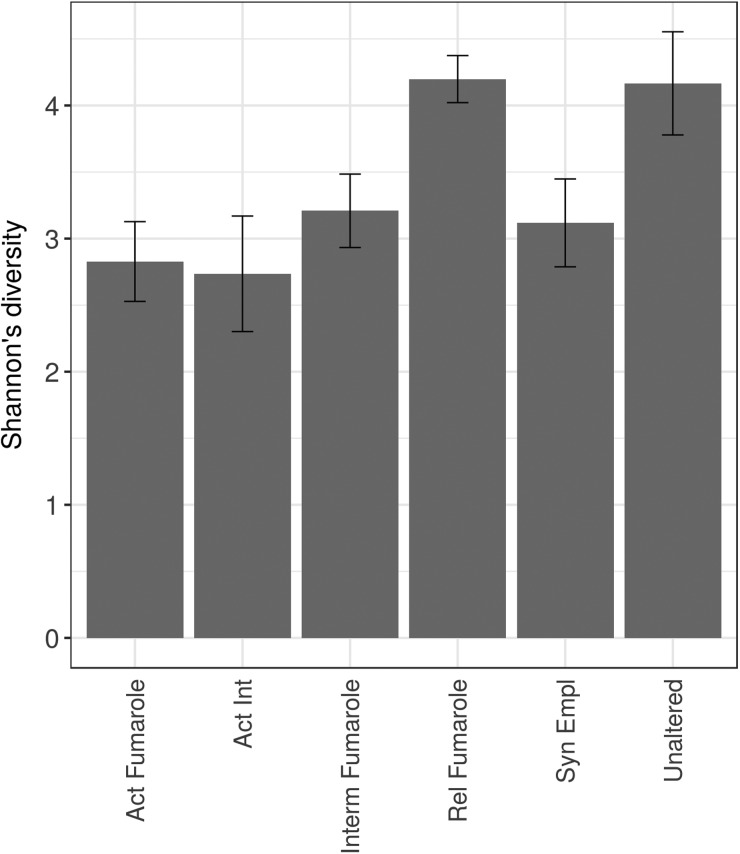
Alpha diversity (Shannon) analysis of the six materials studied. The data are shown as means ± standard error.

Diversity indices were calculated for the materials collected in Hawai‘i to allow us to compare the values to other studies and to carry out a comparison of the diversity between the different materials. The OTU frequencies observed for each of the six material types were in agreement with estimated species richness values, with active and intermediate fumaroles exhibiting lower *Chao1* values (73.75–378.63) than other materials (135.00–738.91) (see Supplementary Fig. S1). Alpha diversity of all the samples was low (Supplementary Fig. S1; see Fig. 2 for means), with Shannon's diversity (*H’*) ranging from 1.84 (active intermediate fumaroles) to 5.09 (unaltered basalt). Shannon's diversity differed between material types (one-way ANOVA, *F*_5,18_ = 3.92, *p* = 0.014) and was generally lower for active and intermediate fumaroles than for other materials (Fig. 2). *Post hoc* Tukey's HSD comparisons of Shannon's diversity between individual alteration types showed near-significant (*p* < 0.05) differences between active intermediate versus relic fumaroles (*p* = 0.052) and active intermediate versus unaltered basalt (*p* = 0.060). Slightly less pronounced differences were observed between active versus relic fumaroles (*p* = 0.077) and active fumaroles versus unaltered basalt (*p* = 0.088).

### 3.3. Microbial community structure and composition in Hawai‘i materials

#### 3.3.1. Bacterial community structure

**FIG. 3.**
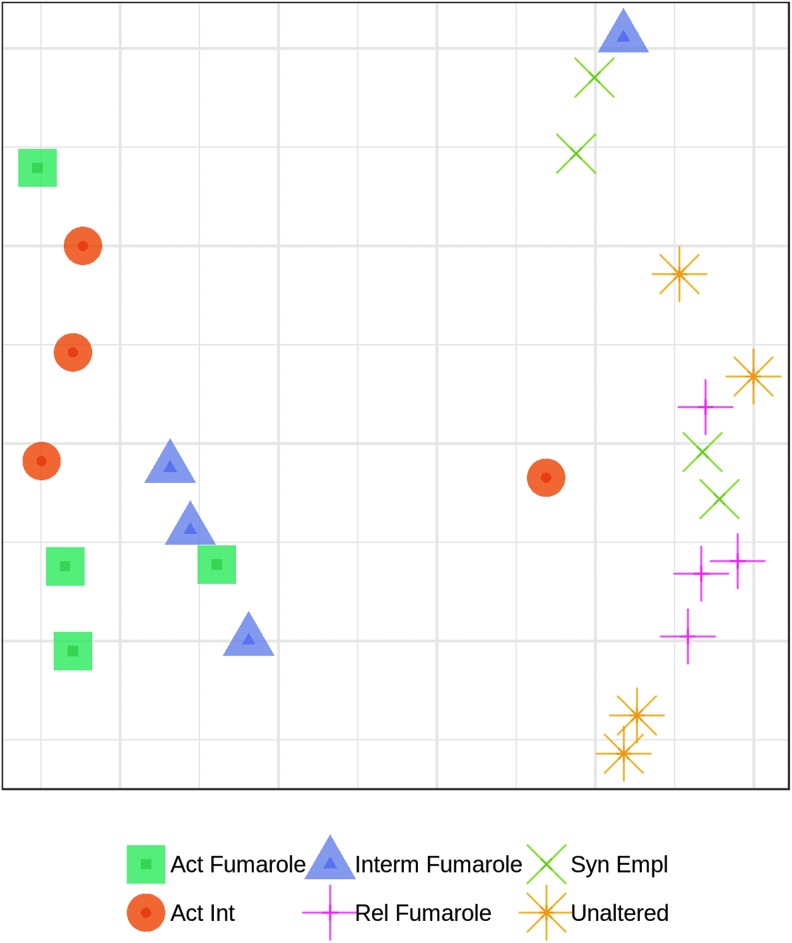
Nonmetric multidimensional scaling (nMDS) ordination of bacterial communities in the six materials studied in Hawai‘i. The ordination was derived from a Bray-Curtis resemblance matrix calculated from untransformed relative OTU abundance data (stress = 0.10).

Fumaroles with active hydrological cycles (active fumaroles, active intermediate and intermediate fumaroles) were found to cluster separately from unaltered, relict, and syn-emplacement material, as shown by a nMDS ordination (Fig. 3).

There was a significant overall influence of material type on bacterial community structure (one-way global PERMANOVA: pseudo-*F*_5,18_ = 2.895, *p* < 0.001). No differences in multivariate dispersion were detected between material types (PERMDISP: *F*_5,18_ = 0.525, *p* = 0.787).

*Post hoc* pairwise comparisons between material types indicated several near-significant (*p* < 0.05) differences between the individual material types (Table 1). However, none of the observed differences were significant at a probability of 0.05.

**Table 1.** Pairwise Comparisons (PERMANOVA) of Bacterial Community Structure in the Six Rock Types from Hawai‘i

	*Active fumarole*	*Active intermediate*	*Intermediate fumarole*	*Relict fumarole*	*Syn emplacement*
Active intermediate	0.138	—	—	—	—
Intermediate fumarole	0.682	0.138	—	—	—
Relict fumarole	0.071	0.071	0.071	—	—
Syn emplacement	0.071	0.071	0.138	0.071	—
Unaltered	0.071	0.103	0.071	0.138	0.138

**FIG. 4.**
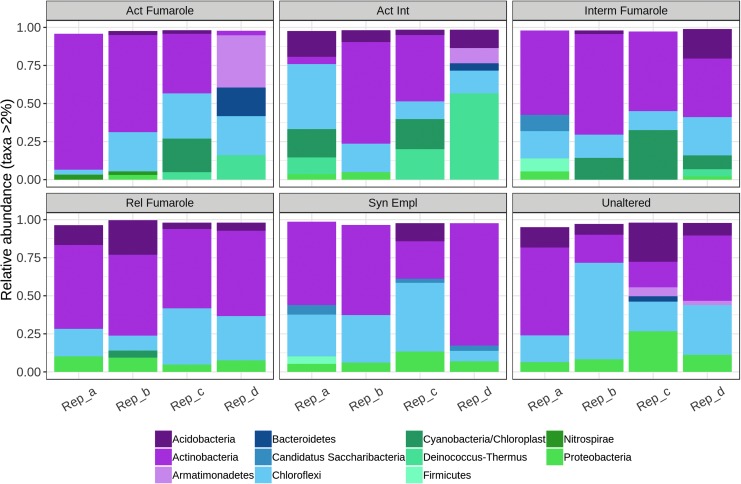
Relative abundances (>2%) of bacterial phyla in the six materials studied in Hawai‘i.

**FIG. 5.**
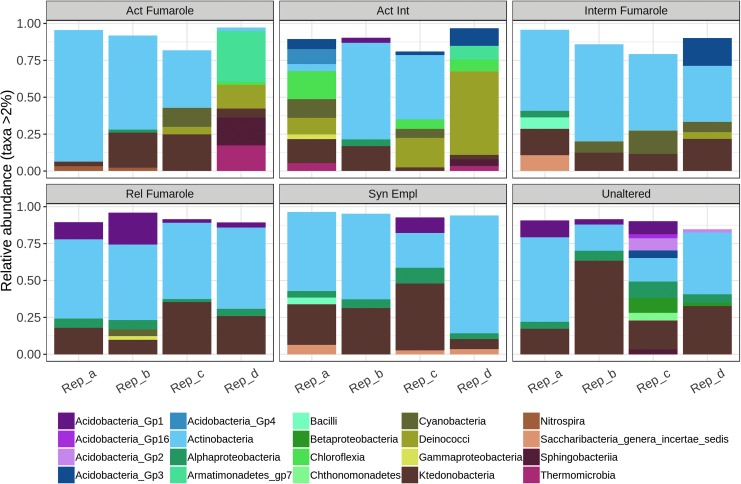
Relative abundances (>2%) of bacterial classes in the six materials studied in Hawai‘i.

The phylum-level analysis across all six material types studied from Hawai‘i is shown in Fig. 4, and at class level it is shown in Fig. 5.

Some taxa reflected the two groupings observed in the nMDS ordination (see Fig. 3). In hydrologically active fumarolic environments, members of the class Deinococci (phylum Deinococcus-Thermus) were more prevalent than in relict or nonfumarolic sites. In the six replicates in which they had a relative abundance higher than 2% (two in active, three in active intermediate, and one in intermediate fumaroles), their abundance ranged from 4.7% (intermediate fumarole, replicate d) to 56.7% (active intermediate fumarole, replicate d) with a mean (standard deviation, s.d.) of 18.9 ± 19.4%. Six of the seven occurrences of Cyanobacteria at higher than 2% relative abundance were encountered in active, active intermediate, or intermediate fumaroles, with a single occurrence in relic fumaroles. Cyanobacterial abundances ranged from 4.6% (relic fumarole, replicate b) to 15.8% (intermediate fumarole, replicate c), with a mean (s.d.) of 9.5 ± 4.2%. A greater abundance of classes within the phylum Proteobacteria was found in relict and nonfumarolic replicates than in hydrologically active fumarolic sites. In the 19 replicates they appeared in with a relative abundance greater than 2%, their abundance ranged from 2.0% (active fumarole, replicate b) to 11% (unaltered, replicate c) with a mean (s.d.) of 5.4 ± 2.7%. They were primarily accounted for by Alphaproteobacteria of the families Bradyrhizobiaceae and Acetobacteraceae (in 15 of the occurrences). Two occurrences were Gammaproteobacteria (Xanthomonadaceae; active intermediate, replicate a, relict fumarole, replicate b) and two Betaproteobacteria (unknown class; unaltered, replicate c and d). Fifteen of the proteobacterial occurrences were in replicates corresponding to unaltered, syn-emplacement, and relict fumaroles (with one occurrence in active, two in active intermediate, and one in intermediate fumaroles). However, despite their wide distribution, the Proteobacteria were not in the top 30 most abundant taxa (Table 2).

**Table 2.** Phylogenetic Affiliations of the 30 Most Abundant Bacterial OTUs within the Hawai‘i Data Set

*OTU*	*Phylogenetic affiliation*
DENOVO33	Chloroflexi; Ktedonobacteria; Thermogemmatisporales; Thermogemmatisporaceae; *Thermogemmatispora*
DENOVO10	Deinococcus-Thermus; Deinococci; Thermales; Thermaceae; *Meiothermus*
DENOVO42	Chloroflexi; Chloroflexia; Chloroflexales; Chloroflexaceae
DENOVO36	Armatimonadetes; Armatimonadetes_gp7; Armatimonadetes_gp7; Armatimonadetes_gp7; Armatimonadetes_gp7
DENOVO21	*Candidatus* Saccharibacteria
DENOVO5	Actinobacteria; Actinobacteria; Actinomycetales
DENOVO27	Actinobacteria; Actinobacteria; Actinomycetales
DENOVO20	Chloroflexi; Ktedonobacteria; Ktedonobacterales; Thermosporotrichaceae; *Thermosporothrix*
DENOVO59	Actinobacteria; Actinobacteria; Actinomycetales; Pseudonocardiaceae
DENOVO56	Actinobacteria; Actinobacteria; Actinomycetales; Pseudonocardiaceae
DENOVO9	Actinobacteria; Actinobacteria; Acidimicrobiales
DENOVO4	Chloroflexi; Ktedonobacteria; Ktedonobacterales; Thermosporotrichaceae; *Thermosporothrix*
DENOVO48	Actinobacteria; Actinobacteria; Actinomycetales; Mycobacteriaceae; *Mycobacterium*
DENOVO37	Acidobacteria; Acidobacteria_Gp1; Gp1; Gp1; Gp1
DENOVO31	Acidobacteria; Acidobacteria_Gp1; *Bryocella; Bryocella; Bryocella*
DENOVO8	Actinobacteria; Actinobacteria; Solirubrobacterales
DENOVO3	Actinobacteria; Actinobacteria; Actinomycetales; Pseudonocardiaceae
DENOVO6	Chloroflexi; Ktedonobacteria; Ktedonobacterales
DENOVO641	Actinobacteria; Actinobacteria; Solirubrobacterales
DENOVO53	Chloroflexi; Ktedonobacteria; Ktedonobacterales
DENOVO23	Actinobacteria; Actinobacteria; Acidimicrobiales
DENOVO47	Chloroflexi; Ktedonobacteria; Ktedonobacterales
DENOVO18	Actinobacteria; Actinobacteria
DENOVO61	Chloroflexi; Ktedonobacteria; Ktedonobacterales; Ktedonobacteraceae; *Ktedonobacter*
DENOVO51	Actinobacteria; Actinobacteria; Acidimicrobiales; Acidimicrobineae_incertae_sedis; *Aciditerrimonas*
DENOVO41	Actinobacteria; Actinobacteria; Actinomycetales
DENOVO15	Chloroflexi; Ktedonobacteria; Ktedonobacterales; Thermosporotrichaceae; *Thermosporothrix*
DENOVO46	Chloroflexi; Ktedonobacteria; Thermogemmatisporales; Thermogemmatisporaceae; *Thermogemmatispora*
DENOVO2	Actinobacteria; Actinobacteria; Actinomycetales
DENOVO16	Cyanobacteria/Chloroplast^*^

**FIG. 6.**
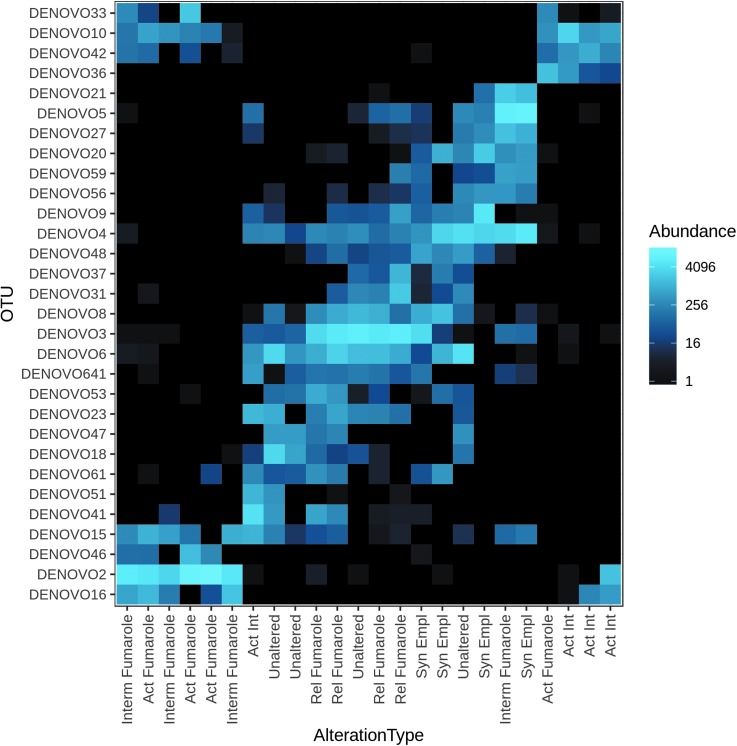
Heat map of top 30 taxa across sample types based on abundance. The taxa identifications are provided in Table 2. The heat map was constructed using raw read number data, with the color scale based on default settings in the phyloseq package (log transformation with base 4).

The OTUs are listed in order to match heat map clustering as shown in Fig. 6. Phylogenetic affiliations are shown as phylum; class; order; family; genus to the highest taxonomic ranking possible.

^*^ Uncultivated cyanobacterium, nearest BLASTn match: Hawai‘i lava tunnel cyanobacterial clone (GenBank ID EF032788.1, 100% similarity).

Some taxa were found to be abundant in replicates across samples and may contribute to the lack of clear pairwise differences between alteration types. Acidobacteria, Actinobacteria, and Chloroflexi were common members of all sample types. Classes within the phylum Acidobacteria ranged from 2.2% (unaltered fumarole, replicate d) to 21.5% (relic fumarole, replicate b) with a mean (s.d.) of 8.1 ± 5.7%. Actinobacterial classes ranged from 2.4% (active fumarole, replicate d) to 89.1% (active fumarole, replicate a) with a mean (s.d.) of 47.1 ± 21.6%. Classes within the phylum Chloroflexi ranged from 2.2% (active fumarole, replicate d) to 63.4% (unaltered, replicate b) with a mean (s.d.) of 17.9 ± 13.8%. The complete data set of taxa with greater than 2% abundance in the samples can be found in Supplementary Table S4.

To further visualize the distribution of bacterial taxa within and between different material types, a heat map analysis was undertaken to show OTU abundance and distribution of the top 30 most abundant taxa in the Hawai‘i data set. These data are shown in Fig. 6. The order of the sample types in the heat map represents the clustering of the samples based on nMDS ordination. This is also the case for the taxa order. The map was based on raw read number data and produced results that were broadly comparable with a map based on taxa relative abundance, with many of the major OTUs being the same (results not shown).

Although classes belonging to the phylum Chloroflexi were found in all material types and relict fumarole and non-fumarolic samples had greater proportions of the class Ktedonobacteria (Fig. 5), the heat map shows that distinct taxa belonging to this phylum were present in active, intermediate, and active intermediate fumaroles and account for the clustering of these samples. These taxa included representatives of the families Thermogemmatisporaceae and Thermosporotrichaceae. In relict fumaroles and nonfumarolic materials, taxa specific to these environments were found in the Actinobacteria (primarily the orders Actinomycetales and Acidimicrobiales). These materials also host representatives of the Acidobacteria (Gp1) (Fig. 5; Table 2) in contrast to the hydrologically active fumarolic environments that primarily host Acidobacteria (Gp3) (Fig. 5), although these latter organisms do not appear in the top 30 taxa in Table 2. While taxa associated with phototrophs were represented in all samples, one uncultured taxon (DENOVO16) associated specifically with fumarolic environments. This OTU was closely related to a cyanobacterial clone previously encountered in Hawaiian lava cave samples (GenBank ID EF032788.1; 100% similarity).

#### 3.3.2. Archaeal community structure

**FIG. 7.**
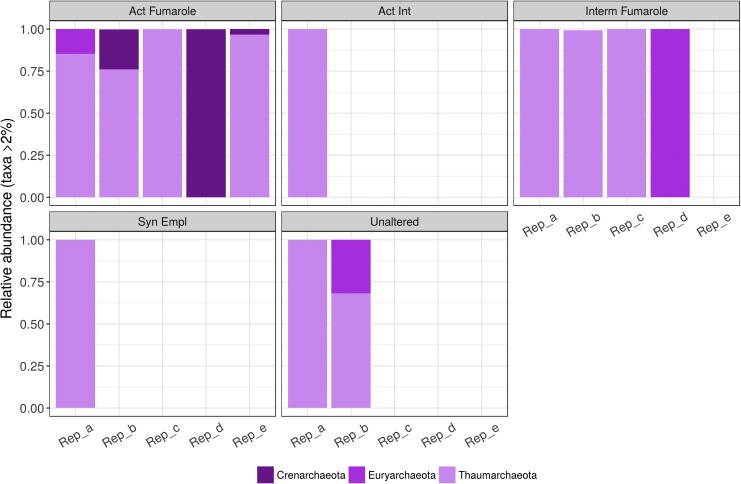
Relative abundances (>2%) of archaeal phyla in the six materials studied in Hawai‘i. Samples with no bars are replicates from which no archaeal DNA was amplified.

The archaeal populations within the materials we examined were found to be of remarkably low diversity. In the Hawai‘i materials, all the active fumarole samples contained archaea that affiliated with Nitrososphaerales (Thaumarchaeota), Thermoplasmata (Euryarchaeota), and Thermoprotei (Crenarchaeota). Identical taxa were found in other materials, but their presence was heterogeneous (Fig. 7). No archaea were recorded in relict fumaroles.

### 3.4. Microbial community structure and composition in Idaho materials

**FIG. 8.**
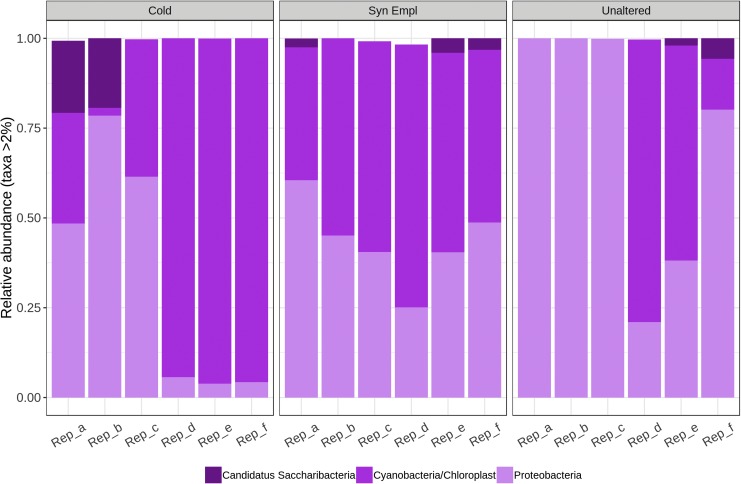
Relative abundances (>2%) of bacterial phyla in the three materials studied in Idaho.

The three materials (unaltered, syn-emplacement, and cold-altered materials) from Idaho were similar to one another in terms of phylogenetic composition (Fig. 8). They had high relative abundances of Alphaproteobacteria, which themselves were dominated by the orders Rhizobiales, Caulobacterales, Sphingomonadales, and Rhodospirillales. High abundances of cyanobacterial sequences were additionally observed. The rocks contained Saccharibacteria, which were also observed in the Hawai‘i materials.

In the Idaho samples, all materials recorded the presence of archaea, but only one taxon was identified. It affiliated with Nitrososphaerales (Thaumarchaeota) (data not shown).

## 4. Discussion

Motivated by an interest in assessing the habitability of extreme environments on Earth with a view to advancing our understanding of the habitability of Mars and how human explorers will eventually explore that world, we carried out a study of basaltic terrains in Hawai‘i with a focus on volcanic fumaroles, localized areas of extensive water-rock interactions. We investigated six materials, four of them associated with various states of aqueous activity within basalt-hosted fumaroles, one of them unaltered basalt, and one of them syn-emplacement basalt.

### 4.1. A low-diversity biota in Hawai‘i basalts and their associated fumaroles

Surprisingly, the phylogenetic and diversity data we observed resulted in a rejection of each of our hypotheses. Our first hypothesis was that basaltic materials in general would host high microbial diversity, as they contain diverse bioavailable elements such as iron, magnesium, and trace elements useful to life such as copper and zinc (Wackett *et al.,* 2004). Our data show that all the materials we studied host a remarkably low-diversity microbial community. The abundance of OTUs, used as a proxy for “species,” ranged from the minimum Chao1 estimate of 73.75 in active fumaroles to a maximum of 738.91 in relict fumaroles. These low values were similarly reflected in the Shannon's diversity index that ranges from 1.84 to 5.09.

Although direct diversity comparisons can be problematic on account of different sequencing platforms and analysis methods, our data are similar to the low diversities recorded in similar environments. Bacterial communities in Icelandic basalts exhibited a Shannon's diversity index of 4.04 (confidence interval 3.88–4.20) at genus level (Kelly *et al.,* 2011). Microbial communities within fresh basaltic lavas in Iceland, 3 and 5 months after eruption, had Shannon's diversity indices as low as 1.4, and all samples were below 3.0 (Kelly *et al.,* 2014). The closest data set to ours was a study of diverse ash and soil environments at the Kilauea volcano, Hawai‘i (Gomez-Alvarez *et al.,* 2006), with Shannon's diversity indices lowest in a 79-year-old lava (2.62 ± 0.02) and highest in a vegetated 210-year-old ash deposit (3.87 ± 0.17). These results can also be compared to hyperarid high-altitude soils of the Central Andes, where Shannon's diversity indices of 4.03–5.15 were reported (Bull *et al.,* 2017), and Antarctic glacial forefields, where values ranged between 3.15 and 4.78 (Bajerski and Wagner, 2013).

The values in our and similar sites contrast to soils where Shannon's diversity indices typically exceeded 6. For example, values from 6.90 to 7.31 were measured in the Loess Plateau, China (Zeng *et al.,* 2016), and values over 5 are typically found in diverse seasonal and tropical forest soils in southwest China (Lan *et al.,* 2017). Even polar desert soils can host communities with Shannon's diversity indices exceeding 8 (McCann *et al.,* 2016).

One explanation for our data is that, in comparison to soils, the rocks we studied are not presented to the biota as fine crushed material that leaches relatively easily, such as in soils. Thus, it might be expected that the availability of nutrients from the rocks is kinetically limited. The biomass and diversity may also be energetically and carbon limited by a low availability of organic carbon flowing into the system. These systems may be nitrogen limited since there are no indigenous nitrogen-containing minerals in the basalts.

### 4.2. Diversity and biomass of life in fumaroles

Our second hypothesis was that active fumaroles would be sites of enhanced microbial diversity since high water availability and the presence of diverse elements in solution would be conducive to life. Interestingly, this scenario was not supported. Of all sample types, active and active intermediate fumaroles had the lowest diversity. Their mean Shannon's diversity indices were 2.83 and 2.74, respectively, while relic fumaroles and unaltered basalt had values of up to 4.20. While these differences were near-significant (*p* < 0.05), one hypothesis to explain the relatively low diversity of bacterial communities within active fumaroles is that their high temperatures might prohibit colonization by certain taxa. High temperatures in geothermal settings are known to restrict microbial diversity compared to more mesophilic environments. Indeed, Sharp *et al.* (2014) found that in geothermal environments peak diversity occurred at 24°C.

Multivariate ordination of the data showed that active, active intermediate, and intermediate fumaroles generally clustered together and were separated from relict fumaroles and syn-emplacement and unaltered material. Phylogenetic analyses showed that in the more active fumarolic sites a variety of taxa are associated with known thermophiles, including members of the class Ktedonobacteria. This class of bacteria has previously been found in geothermally heated environments (Yabe *et al.,* 2016) and has been shown to occur within geothermal biofilms associated with carbon monoxide metabolism (King and King, 2014). Taxa belonging to Chloroflexi have previously been reported from steam fumaroles in Hawai‘i (Wall *et al.,* 2015) and fumarolic soils in Antarctica (Soo *et al.,* 2009).

In terms of biomass, active fumaroles did have a higher mean biomass than active intermediate and intermediate materials but a lower mean biomass than relict fumaroles and syn-emplacement materials. While based on a qualitative comparison, these data suggest that active fumaroles do not necessarily host higher biomass than less hydrologically active environments. However, some active fumarole samples did yield high concentrations of DNA, accounting for the higher mean and standard deviation compared to other samples. One of the samples from an active fumarole had the highest DNA yield of all samples analyzed, suggesting that active fumaroles harbor microenvironments where conditions of water flow and suitable energy and nutrient fluxes allow for high biomass. The heterogeneous distribution of biomass in basaltic terrains has been noted previously in both fresh lava (Kelly *et al.,* 2014) and older rocks (Gentry *et al.,* 2017). One factor that might account for this is the effect of spatial scale on physicochemical factors. At small scales, variations in fractures, water availability through fractures, porosity, distribution of carbon, and mineral elements available for redox couples could all affect the distribution of the microbial communities within the rocks, as they do soils (Garcia-Pichel *et al.,* 2003; Vos *et al.,* 2013; O'Brien *et al.,* 2016). These data emphasize the important influence of spatial scale, and thus potentially sample size, in determinations on microbial community and biomass distribution.

The explanation for the rejection of the second hypothesis would also account for why our third hypothesis, that diversity and biomass would decline as fumaroles transition from active to relict states, was not supported. As fumaroles transition to a relict state and continuous aqueous activity is lost, the potential for bioavailable minerals released in flowing water is reduced, but this may be more than offset by the less extreme temperature conditions that favor higher diversity, explaining the higher average alpha diversity in relict fumaroles compared to the active fumarolic environments and their similar biomass.

### 4.3. Diversity and biomass of life in unaltered basalts

For these same reasons, the fourth hypothesis, that unaltered basalts would harbor the lowest diversity, was not supported. However, despite revealing the second-highest diversity of our samples (with a mean Shannon's diversity index of 4.17), the unaltered basalts did have low, albeit highly variable, biomass estimates, which may reflect the low water flow and nutrient accessibility in the materials. Although organisms in the unaltered basalt do not require the specialisms associated with higher-temperature active fumaroles, the biomass may be less because of lower fluid flow through the unaltered basalts. The higher inferred biomass observed in syn-emplacement rocks might be caused by the weathered texture of the rocks compared to the more impermeable unaltered rocks, which may support higher biomass communities.

### 4.4. Patterns of microbial diversity

One potential interference with these data is that we were not able to differentiate between active organisms in the rocks and those that had drifted into the rocks from the atmosphere and are inactive. We cannot rule out such a population. As shown in the heat map, there are a number of taxa found across the different material types, for example members of the Chloroflexi (Ktedonobacteria) and Actinobacteria. However, both global and pairwise PERMANOVA were indicative of differences between material types, particularly between active fumarolic sites and other environments. Whether all taxa are active in all environments or whether some taxa are distributed across the region, for example in fumarolic emissions or eolian transport, and are inactive requires further investigation.

The bacterial communities in Idaho were structurally and compositionally different from those in Hawai‘i. Alphaproteobacteria dominated the community in comparison to the high abundances of Actinobacteria and Acidobacteria in the Hawaiian materials. These results can be compared to other environments, such as Iceland. An investigation of basaltic rocks of ∼2 ka age in Iceland showed the rocks to be dominated by Acidobacteria (42.1%) and Actinobacteria (15.8%). Acidobacteria were dominated by Gp4 sequences (82%), with class Gp3 comprising the greater proportion of the remaining Acidobacteria (Kelly *et al.,* 2011). Both Gp3 and 4 are found in the Hawaiian materials. Actinobacteria were also found to be an abundant phylum in Icelandic volcanic basaltic and felsic glasses with a high proportion of Acidobacteria (Kelly *et al.,* 2010).

These data suggest that although the specific taxonomic composition of the microbial communities in basaltic rocks may be geographically influenced, the rocks host representatives of phyla that are well adapted for the volcanic rock environment, particularly among the Actinobacteria and Acidobacteria. The pervasiveness of Actinobacteria in many volcanic environments and a laboratory demonstration of their capacity to actively weather volcanic rocks has been reported previously (Cockell *et al.,* 2013). They have also been reported in fumarolic soils (Costello *et al.,* 2009; Soo *et al.,* 2009), potentially as metabolizers of fumarolic gases such as carbon monoxide (Solon *et al.,* 2018).

The high Alphaproteobacteria abundance in the Idaho rocks is primarily accounted for by taxa such as Rhizobiales, Caulobacterales*,* Sphingomonadales*,* and Rhodospirillales that are known inhabitants of soils (*e.g.,* Kuykendall *et al.,* 2005; Garrity *et al.,* 2015). This might reflect microbial input from relatively fertile soils around the Idaho lava flows. Another factor underlying the abundance of these taxa could be their generalist characteristics and ability to use diverse carbon sources (*e.g.,* Kuykendall *et al.,* 2005; Garrity *et al.,* 2015).

A study of the microbial inhabitants of 300-year-old rainforest soil on the Kilauea volcano, Hawai‘i, found it to be dominated by the same taxa as we observed in Idaho: Alphaproteobacteria, primarily accounted for by Rhizobiales, Caulobacterales*,* Sphingomonadales, and Rhodospirillales (Gomez-Alvarez *et al.,* 2007). However, in the same study, the phylogenetic investigation of more recent ash deposits and 79-year-old lava showed a high abundance of Actinobacteria and Acidobacteria (Gomez-Alvarez *et al.,* 2007), consistent with our own investigations in Hawai‘i. One interpretation of these data might be that basaltic rocks host soil-like communities either when the rocks are weathered through to soils, as seems to occur in Hawai‘i, or when they are influenced by microbial input from nearby soils, as may be the case in Idaho.

A potential role for geographical or other physicochemical factors in determining the inhabitants of volcanic materials is suggested by the prevalence of different phyla in other fumarolic sites. For example, abundant Verrucomicrobia were reported in warm fumarolic soils in the Andes (Costello *et al.,* 2009), a phylum for which we only observed one OTU in our samples.

We also found a low diversity of archaeal residents in both Hawai‘i and Idaho. This may reflect a similar functional role for a very limited number of taxa in this domain, in other words a generally restricted niche for archaea in the rocks. The presence of Thermoprotei (Crenarchaeota) is consistent with earlier reports of Crenarchaeota in Hawaiian steam fumaroles (Wall *et al.,* 2015) and other fumaroles (Benson *et al.,* 2011). In both Hawai‘i and Idaho, we observed members of the Nitrososphaerales (Thaumarchaeota), an order of archaea known to be involved in ammonia oxidation (Tourna *et al.,* 2011) and previously reported in geothermal environments (Daebeler *et al.,* 2018). These data suggest a role for the low-diversity archaea in nitrogen cycling.

Although caution should be taken in making functional inferences from 16S rRNA gene sequence data, apart from the phototrophs, the organisms within our samples primarily affiliate with heterotrophic taxa, suggesting that they get their carbon from phototrophs, atmospheric carbon, or necromass. These data further support the idea that the majority of the taxa are using the rocks as a surface rather than as an energy supply. Although some nutrients such as iron may well come from the rock, most organisms are likely sustained by a carbon cycle occurring between community members. This hypothesis would explain why we observe community similarity between rock types such as relict fumaroles, unaltered and syn-emplacement basalts, despite different weathering and aqueous histories. If the rocks are primarily a surface to grow on, then the underlying rock geochemistry in extreme environments with low water flow may have little influence on microbial community composition.

### 4.5. Implications for habitability and the exploration of Mars

One surprising aspect of the rejection of our hypotheses is that the low diversity and biomass we measured is in apparent contradiction to the availability of redox couples for energy acquisition. The basaltic rocks studied here contain ∼8–16% iron oxide, mostly as FeO that is easily oxidized to Fe_2_O_3_. Thus, in combination with atmospheric oxygen as an electron acceptor, this redox couple alone would be expected to fuel a substantial iron-oxidizing community. No sequences affiliated with known iron-oxidizing taxa appear in our analysis. One explanation for these data is that when locked into minerals, many of the potential redox enabling elements and nutrients within basaltic rock and their alteration products are kinetically difficult to access by a biota (Cockell *et al.,* 2011). Additionally, the heterogeneous distribution of these elements at micron scales might limit the extent to which organisms within the rock can access them. These data show that the mere presence of electron donors and acceptors and other nutrients in a bulk sample is not adequate to assess habitability, on Earth or Mars, and may overestimate the biological potential of an environment when considered from a purely thermodynamic standpoint. As we suggest here, even when water flow is high, other physical factors associated with some environments that have high water flow, such as high temperatures, may limit the biota, such that when kinetic factors are taken into account even they cannot, in themselves, predict the biomass or diversity of life that an environment can sustain. These points underscore the more general observation that the habitability of environments cannot be assessed from single factors and that multiple energetic, kinetic, and physical factors must be considered.

Our results have implications for the habitability of similar environments on Mars. If such low diversity is sustained in basaltic terrains on Earth where there is substantial carbon flux through meteoric water and the atmosphere, transiently wet basaltic terrains on Mars may host even less biological potential. Mars receives an estimated organic flux from meteoritic material of 2.4 × 10^6^ kg/year (Flynn, 1996), about 6 orders of magnitude lower than primary productivity on present-day Earth (1.05 × 10^14^ kg C per year; Field *et al.,* 1998). As Mars transitioned into drier conditions with less vigorous hydrology, many of its basaltic environments may have become uninhabitable as the kinetic barriers to elements within rocks made these rocks unsuitable places for life and additional stressors not experienced in the basaltic terrains we studied, including high ionizing radiation flux (Dartnell *et al.,* 2007), ultraviolet radiation (Cockell *et al.,* 2000), and perchlorate-containing salts (Hecht *et al.,* 2009), influenced their physical and chemical environments.

Finally, these data inform strategies for the robotic and human exploration of Mars. The low-diversity, low-biomass communities we find in the materials we studied show that a comprehensive mapping of habitable conditions on Mars at planetary scale down to centimeter scale requires a widespread sampling effort to investigate a large number of samples, particularly those in places where extreme conditions may place them at the limits of habitability.

The heterogeneous distribution of biomass and diversity that we observed, similarly to that observed elsewhere in basaltic rocks (Kelly *et al.,* 2014; Gentry *et al.,* 2017) or soils (Garcia-Pichel *et al.,* 2003; Vos *et al.,* 2013; O'Brien *et al.,* 2016) confirms the need for a large sample suite to maximize the chances of obtaining the right samples. We note that whether a specific sample contains high or low biomass may not be *a priori* predictable merely based on bulk composition or geological context. Despite similar weathering types and having been obtained from the same field site, our samples showed a very wide diversity of biomass and diversity. Although robotic exploration and sample return will significantly advance our understanding of martian environmental conditions, as with the investigations reported here in BASALT, the most effective way to generate a coherent synthetic picture of habitability on Mars at all scales may well be to carry out a sustained program of human exploration and large-scale sampling.

## Supplementary Material

Supplemental data

## Abbreviations Used

ANOVA: analysis of variance
BASALT: Biologic Analog Science Associated with Lava Terrains
EVA: extravehicular activity
HSD: Honest Significant Difference
nMDS: nonmetric multidimensional scaling
OTU: operational taxonomic unit
PERMANOVA: permutational analysis of variance
PERMDISP: test for the homogeneity of multivariate dispersions
s.d.: standard deviation

## Notes

Associate Editor: Victor Parro
