# Supplementary material for: A Low-Diversity Microbiota Inhabits Extreme Terrestrial Basaltic Terrains and Their Fumaroles: Implications for the Exploration of Mars
Source: Astrobiology. 2019 Mar 6;19(3):284–99. doi: 10.1089/ast.2018.1870 (PMC6442273; doi:10.1089/ast.2018.1870)

Supplementary Information

Supplementary Methods

Table S1A. Location of samples acquired in the study from Hawai'i.

Sample type	Sample number	Location (lat, long)
Unaltered	113 (replicate a)	19.36889498<br>-155.1983918
Unaltered	603 (replicate b)	19.36608283<br>-155.2005604
Unaltered	689 (replicate c)	19.36607949<br>-155.2005505
Unaltered	690 (replicate d)	19.36608499<br>-155.2005614
Syn-emplacement	104 (replicate a)	19.36861544<br>-155.2001828
Syn-emplacement	105 (replicate b)	19.36861544<br>-155.2001828
Syn-emplacement	612 (replicate c)	19.36788826<br>-155.1992048
Syn-emplacement	615 (replicate d)	19.36788224<br>-155.1992092
Relict fumaroles	604 (replicate a)	19.36803954<br>-155.1992317
Relict fumaroles	605 (replicate b)	19.36803956<br>-155.1992284
Relict fumaroles	698 (replicate c)	19.36686542<br>-155.2009675
Relict fumaroles	701 (replicate d)	19.36688885<br>-155.2009939
Intermediate fumaroles	91 (replicate a)	19.36466235<br>-155.2093681
Intermediate fumaroles	98 (replicate b)	19.36467504<br>-155.2093575
Intermediate fumaroles	99 (replicate c)	19.36466203<br>-155.2093675
Intermediate fumaroles	707 (replicate d)	19.36822158<br>-155.2002675
Active intermediate fumaroles	85 (replicate a)	19.36465895<br>-155.2094187
Active intermediate fumaroles	90 (replicate b)	19.36466125<br>-155.2094183
Active intermediate fumaroles	132 (replicate c)	19.3682095<br>-155.2002712
Active intermediate fumaroles	723 (replicate d)	19.36819705<br>-155.2002845
Active fumaroles	63 (replicate a)	19.36435432<br>-155.2092684
Active fumaroles	65 (replicate b)	19.36435558<br>-155.2092652
Active fumaroles	84 (replicate c)	19.36434905<br>-155.2092626
Active fumaroles	86 (replicate d)	19.36560004<br>-155.2058711

Table S1B. Location of samples acquired in the study from Idaho.

Sample type	Sample number	Location (lat, long)
Unaltered	250 (replicate a)	43.46419247<br>-113.5701232
Unaltered	251 (replicate b)	43.46419247<br>-113.5701232
Unaltered	447 (replicate c)	43.46437139<br>-113.5715804
Unaltered	460 (replicate d)	43.44584263<br>-113.5658088
Unaltered	470 (replicate e)	43.46436154<br>-113.5715575
Unaltered	105 (replicate f)	43.44352873<br>-113.563933
Syn-emplacement	307 (replicate a)	43.4636149<br>-113.571957
Syn-emplacement	308 (replicate b)	43.46360364<br>-113.5717956
Syn-emplacement	448 (replicate c)	43.46362648<br>-113.5719494
Syn-emplacement	441 (replicate d)	43.46406218<br>-113.5719145
Syn-emplacement	442 (replicate e)	43.46404875<br>-113.5719251
Syn-emplacement	439 (replicate f)	43.4640628<br>-113.571915
Cold altered	198 (replicate a)	43.44548071<br>-113.5657901
Cold altered	210 (replicate b)	43.44548071<br>-113.5657901
Cold altered	209 (replicate c)	43.44548071<br>-113.5657901
Cold altered	271 (replicate d)	43.46272771<br>-113.5691469
Cold altered	261 (replicate e)	43.46273488<br>-113.5690713
Cold altered	258 (replicate f)	43.46268522<br>-113.569077

Table S2. Data processing parameters for bacterial and archaeal sequences (implemented in micca v. 1.6.2; http://micca.org/). The sequence lengths are shown in the micca filter row (Bacterial sequences: Hawaii (277), Idaho (275); Archaeal sequences: Hawaii (308), Idaho (308)).

	**Bacterial sequences**		**Archaeal sequences**	
**Command**	Hawaii	Idaho	Hawaii	Idaho
micca mergepairs	-l 175 -d 50	-l 175 -d 50	-l 150 -d 50	-l 150 -d 50
micca filter	-e 0.25 -m 277<br>(90.5% of reads retained)	-e 0.25 -m 275<br>(85.1% of reads retained)	-e 0.25 -m 308<br>(90.6% of reads retained)	-e 0.25 -m 308<br>(88.3% of reads retained)

Supplementary Results

Table S3. Sample type, number, raw DNA concentration and estimated biomass for all samples. The samples in bold are those that were used for sequence analysis. The 'sample number' corresponds to sequential samples in the figures of the main text and their rarefaction curves are shown in Figure S2.

Sample Type	Sample Number	DNA concentration (µg/g dry weight)	Estimated biomass (cells/g dry weight)
**Active Fumarole**	**63**	**0.022**	**13750000**
**Active Fumarole**	**65**	**0.003**	**1875000**
**Active Fumarole**	**84**	**0.077**	**48125000**
**Active Fumarole**	**86**	**0.001**	**625000**
Active Fumarole	95	0.047	29380000
Active Fumarole	76	0.033	20630000
**Active Intermediate**	**85**	**0.001**	**625000**
**Active Intermediate**	**90**	**0.006**	**3750000**
**Active Intermediate**	**132**	**0.001**	**625000**
**Active Intermediate**	**723**	**0.001**	**625000**
Active Intermediate	80	0	0
Active Intermediate	131	0	0
**Intermediate fumarole**	**91**	**0.003**	**1875000**
**Intermediate fumarole**	**98**	**0.023**	**14375000**
**Intermediate fumarole**	**99**	**0.013**	**8125000**
**Intermediate fumarole**	**707**	**0.001**	**625000**
Intermediate fumarole	712	0.001	625000
Intermediate fumarole	129	0.002	1250000
**Relict fumarole**	**604**	**0.054**	**33750000**
**Relict fumarole**	**605**	**0.034**	**21250000**
**Relict fumarole**	**698**	**0.022**	**13750000**
**Relict fumarole**	**701**	**0.028**	**17500000**

Relict fumarole	608	0.034	21250000
Relict fumarole	703	0.035	21880000
**Syn Emplacement**	**104**	**0.008**	**5000000**
**Syn Emplacement**	**105**	**0.005**	**3125000**
**Syn Emplacement**	**612**	**0.059**	**36875000**
**Syn Emplacement**	**615**	**0.013**	**8125000**
Syn Emplacement	103	0.007	4375000
Syn Emplacement	610	0.065	40630000
**Unaltered**	**113**	**0.043**	**26875000**
**Unaltered**	**603**	**0.001**	**625000**
**Unaltered**	**689**	**0.003**	**1875000**
**Unaltered**	**690**	**0.004**	**2500000**
Unaltered	107	0.036	22500000
Unaltered	109	0.002	1250000

Table S4. Taxa with higher than 2% relative abundance in the Hawaii materials. The OTU list and abundance values are based on data agglomerated at the Class level.

**OTU**	**Abundance**	**Material**	**Samples**	**Phylum**	**Class**
DENOVO2	0.89163300	Act Fumarole	ActFum_a	Actinobacteria	Actinobacteria
DENOVO2	0.79900887	Syn Empl	Syn_d	Actinobacteria	Actinobacteria
DENOVO2	0.65824815	Interm Fumarole	Inter_b	Actinobacteria	Actinobacteria
DENOVO2	0.65445462	Act Int	ActInt_b	Actinobacteria	Actinobacteria
DENOVO2	0.63792211	Act Fumarole	ActFum_b	Actinobacteria	Actinobacteria
DENOVO4	0.63430681	Unaltered	Un_b	Chloroflexi	Ktedonobacteria
DENOVO2	0.58010677	Syn Empl	Syn_b	Actinobacteria	Actinobacteria
DENOVO2	0.57276543	Unaltered	Un_a	Actinobacteria	Actinobacteria
DENOVO10	0.56611879	Act Int	ActInt_d	Deinococcus-Thermus	Deinococci
DENOVO2	0.55058646	Rel Fumarole	Rel_d	Actinobacteria	Actinobacteria
DENOVO2	0.54846102	Interm Fumarole	Inter_a	Actinobacteria	Actinobacteria
DENOVO2	0.53793932	Rel Fumarole	Rel_a	Actinobacteria	Actinobacteria
DENOVO2	0.53630913	Syn Empl	Syn_a	Actinobacteria	Actinobacteria
DENOVO2	0.51863765	Interm Fumarole	Inter_c	Actinobacteria	Actinobacteria
DENOVO2	0.51691206	Rel Fumarole	Rel_c	Actinobacteria	Actinobacteria
DENOVO2	0.51233616	Rel Fumarole	Rel_b	Actinobacteria	Actinobacteria

DENOVO4	0.45246395	Syn Empl	Syn_c	Chloroflexi	Ktedonobacteria
DENOVO2	0.43562232	Act Int	ActInt_c	Actinobacteria	Actinobacteria
DENOVO2	0.41919704	Unaltered	Un_d	Actinobacteria	Actinobacteria
DENOVO2	0.39028724	Act Fumarole	ActFum_c	Actinobacteria	Actinobacteria
DENOVO2	0.37870166	Interm Fumarole	Inter_d	Actinobacteria	Actinobacteria
DENOVO4	0.35296458	Rel Fumarole	Rel_c	Chloroflexi	Ktedonobacteria
DENOVO36	0.34338220	Act Fumarole	ActFum_d	Armatimonadetes	Armatimonadetes_gp7
DENOVO4	0.32601563	Unaltered	Un_d	Chloroflexi	Ktedonobacteria
DENOVO4	0.31226606	Syn Empl	Syn_b	Chloroflexi	Ktedonobacteria
DENOVO4	0.27469187	Syn Empl	Syn_a	Chloroflexi	Ktedonobacteria
DENOVO4	0.25817957	Rel Fumarole	Rel_d	Chloroflexi	Ktedonobacteria
DENOVO4	0.24903761	Act Fumarole	ActFum_c	Chloroflexi	Ktedonobacteria
DENOVO4	0.23781856	Act Fumarole	ActFum_b	Chloroflexi	Ktedonobacteria
DENOVO2	0.23601648	Syn Empl	Syn_c	Actinobacteria	Actinobacteria
DENOVO4	0.21728022	Interm Fumarole	Inter_d	Chloroflexi	Ktedonobacteria
DENOVO31	0.21462992	Rel Fumarole	Rel_b	Acidobacteria	Acidobacteria_Gp1
DENOVO10	0.20037554	Act Int	ActInt_c	Deinococcus-Thermus	Deinococci
DENOVO4	0.19534422	Unaltered	Un_c	Chloroflexi	Ktedonobacteria
DENOVO42	0.18986486	Act Int	ActInt_a	Chloroflexi	Chloroflexia
DENOVO96	0.18847985	Interm Fumarole	Inter_d	Acidobacteria	Acidobacteria_Gp3
DENOVO69	0.18748709	Act Fumarole	ActFum_d	Bacteroidetes	Sphingobacteriia
DENOVO4	0.17993391	Rel Fumarole	Rel_a	Chloroflexi	Ktedonobacteria
DENOVO4	0.17915075	Interm Fumarole	Inter_a	Chloroflexi	Ktedonobacteria
DENOVO2	0.17737496	Unaltered	Un_b	Actinobacteria	Actinobacteria
DENOVO63	0.17427215	Act Fumarole	ActFum_d	Chloroflexi	Thermomicrobia
DENOVO4	0.17298094	Unaltered	Un_a	Chloroflexi	Ktedonobacteria
DENOVO4	0.16864152	Act Int	ActInt_b	Chloroflexi	Ktedonobacteria
DENOVO4	0.16283784	Act Int	ActInt_a	Chloroflexi	Ktedonobacteria
DENOVO10	0.16023126	Act Fumarole	ActFum_d	Deinococcus-Thermus	Deinococci

DENOVO2	0.15871154	Unaltered	Un_c	Actinobacteria	Actinobacteria
DENOVO81	0.15799432	Interm Fumarole	Inter_c	Cyanobacteria/Chloroplast	Cyanobacteria
DENOVO81	0.12888659	Act Fumarole	ActFum_c	Cyanobacteria/Chloroplast	Cyanobacteria
DENOVO81	0.12747748	Act Int	ActInt_a	Cyanobacteria/Chloroplast	Cyanobacteria
DENOVO4	0.12457406	Interm Fumarole	Inter_b	Chloroflexi	Ktedonobacteria
DENOVO96	0.11878969	Act Int	ActInt_d	Acidobacteria	Acidobacteria_Gp3
DENOVO4	0.11617786	Interm Fumarole	Inter_c	Chloroflexi	Ktedonobacteria
DENOVO31	0.11504956	Rel Fumarole	Rel_a	Acidobacteria	Acidobacteria_Gp1
DENOVO31	0.11405399	Unaltered	Un_a	Acidobacteria	Acidobacteria_Gp1
DENOVO10	0.11148649	Act Int	ActInt_a	Deinococcus-Thermus	Deinococci
DENOVO60	0.11043941	Unaltered	Un_c	Proteobacteria	Alphaproteobacteria
DENOVO21	0.10707706	Interm Fumarole	Inter_a	Candidatus Saccharibacteria	Saccharibacteria_genera_incertae_sedis
DENOVO60	0.10584693	Syn Empl	Syn_c	Proteobacteria	Alphaproteobacteria
DENOVO31	0.10505467	Syn Empl	Syn_c	Acidobacteria	Acidobacteria_Gp1
DENOVO302	0.10270270	Act Int	ActInt_a	Acidobacteria	Acidobacteria_Gp4
DENOVO164	0.10096544	Unaltered	Un_c	Proteobacteria	Betaproteobacteria
DENOVO4	0.09830378	Rel Fumarole	Rel_b	Chloroflexi	Ktedonobacteria
DENOVO36	0.09264102	Act Int	ActInt_d	Armatimonadetes	Armatimonadetes_gp7
DENOVO31	0.08833348	Unaltered	Un_c	Acidobacteria	Acidobacteria_Gp1
DENOVO185	0.08364161	Unaltered	Un_c	Acidobacteria	Acidobacteria_Gp2
DENOVO42	0.08012701	Act Int	ActInt_d	Chloroflexi	Chloroflexia
DENOVO83	0.07737587	Interm Fumarole	Inter_a	Firmicutes	Bacilli
DENOVO81	0.07556625	Interm Fumarole	Inter_b	Cyanobacteria/Chloroplast	Cyanobacteria
DENOVO81	0.06979445	Interm Fumarole	Inter_d	Cyanobacteria/Chloroplast	Cyanobacteria
DENOVO4	0.06811110	Syn Empl	Syn_d	Chloroflexi	Ktedonobacteria
DENOVO60	0.06760658	Unaltered	Un_b	Proteobacteria	Alphaproteobacteria
DENOVO96	0.06756757	Act Int	ActInt_a	Acidobacteria	Acidobacteria_Gp3
DENOVO42	0.06384120	Act Int	ActInt_c	Chloroflexi	Chloroflexia
DENOVO21	0.06316622	Syn Empl	Syn_a	Candidatus Saccharibacteria	Saccharibacteria_genera_incertae_sedis

DENOVO60	0.06240362	Rel Fumarole	Rel_b	Proteobacteria	Alphaproteobacteria
DENOVO60	0.06182037	Rel Fumarole	Rel_a	Proteobacteria	Alphaproteobacteria
DENOVO4	0.06153211	Act Fumarole	ActFum_d	Chloroflexi	Ktedonobacteria
DENOVO81	0.06142704	Act Int	ActInt_c	Cyanobacteria/Chloroplast	Cyanobacteria
DENOVO60	0.06044057	Syn Empl	Syn_b	Proteobacteria	Alphaproteobacteria
DENOVO60	0.05661278	Unaltered	Un_d	Proteobacteria	Alphaproteobacteria
DENOVO63	0.05337838	Act Int	ActInt_a	Chloroflexi	Thermomicrobia
DENOVO215	0.05296400	Unaltered	Un_c	Armatimonadetes	Chthonomonadetes
DENOVO96	0.05097898	Unaltered	Un_c	Acidobacteria	Acidobacteria_Gp3
DENOVO10	0.04982233	Act Fumarole	ActFum_c	Deinococcus-Thermus	Deinococci
DENOVO60	0.04945470	Rel Fumarole	Rel_d	Proteobacteria	Alphaproteobacteria
DENOVO60	0.04713022	Unaltered	Un_a	Proteobacteria	Alphaproteobacteria
DENOVO10	0.04668447	Interm Fumarole	Inter_d	Deinococcus-Thermus	Deinococci
DENOVO2	0.04639640	Act Int	ActInt_a	Actinobacteria	Actinobacteria
DENOVO83	0.04617755	Syn Empl	Syn_a	Firmicutes	Bacilli
DENOVO81	0.04582691	Rel Fumarole	Rel_b	Cyanobacteria/Chloroplast	Cyanobacteria
DENOVO60	0.04528278	Act Int	ActInt_b	Proteobacteria	Alphaproteobacteria
DENOVO69	0.04445275	Act Int	ActInt_d	Bacteroidetes	Sphingobacteriia
DENOVO60	0.04443945	Interm Fumarole	Inter_a	Proteobacteria	Alphaproteobacteria
DENOVO60	0.04413724	Syn Empl	Syn_a	Proteobacteria	Alphaproteobacteria
DENOVO60	0.03895356	Syn Empl	Syn_d	Proteobacteria	Alphaproteobacteria
DENOVO31	0.03571668	Unaltered	Un_b	Acidobacteria	Acidobacteria_Gp1
DENOVO31	0.03471466	Act Int	ActInt_b	Acidobacteria	Acidobacteria_Gp1
DENOVO31	0.03457027	Rel Fumarole	Rel_d	Acidobacteria	Acidobacteria_Gp1
DENOVO21	0.03445891	Syn Empl	Syn_d	Candidatus Saccharibacteria	Saccharibacteria_genera_incertae_sedis
DENOVO63	0.03399328	Act Int	ActInt_d	Chloroflexi	Thermomicrobia
DENOVO69	0.03320401	Unaltered	Un_c	Bacteroidetes	Sphingobacteriia
DENOVO90	0.03277727	Act Fumarole	ActFum_a	Nitrospirae	Nitrospira
DENOVO113	0.03265766	Act Int	ActInt_a	Proteobacteria	Gammaproteobacteria

DENOVO4	0.03127851	Act Fumarole	ActFum_a	Chloroflexi	Ktedonobacteria
DENOVO4	0.03119163	Act Int	ActInt_d	Chloroflexi	Ktedonobacteria
DENOVO137	0.02724894	Unaltered	Un_c	Acidobacteria	Acidobacteria_Gp16
DENOVO21	0.02693709	Syn Empl	Syn_c	Candidatus Saccharibacteria	Saccharibacteria_genera_incertae_sedis
DENOVO113	0.02549152	Rel Fumarole	Rel_b	Proteobacteria	Gammaproteobacteria
DENOVO96	0.02414163	Act Int	ActInt_c	Acidobacteria	Acidobacteria_Gp3
DENOVO4	0.02387339	Act Int	ActInt_c	Chloroflexi	Ktedonobacteria
DENOVO2	0.02374561	Act Fumarole	ActFum_d	Actinobacteria	Actinobacteria
DENOVO31	0.02357740	Rel Fumarole	Rel_c	Acidobacteria	Acidobacteria_Gp1
DENOVO164	0.02326553	Unaltered	Un_d	Proteobacteria	Betaproteobacteria
DENOVO90	0.02237819	Act Fumarole	ActFum_b	Nitrospirae	Nitrospira
DENOVO185	0.02237070	Unaltered	Un_d	Acidobacteria	Acidobacteria_Gp2
DENOVO42	0.02168078	Act Fumarole	ActFum_d	Chloroflexi	Chloroflexia
DENOVO60	0.02079188	Rel Fumarole	Rel_c	Proteobacteria	Alphaproteobacteria
DENOVO60	0.02001956	Act Fumarole	ActFum_b	Proteobacteria	Alphaproteobacteria

Figure S1. Observed, Chao1 and Shannon diversity index data for the six different Hawaii materials.

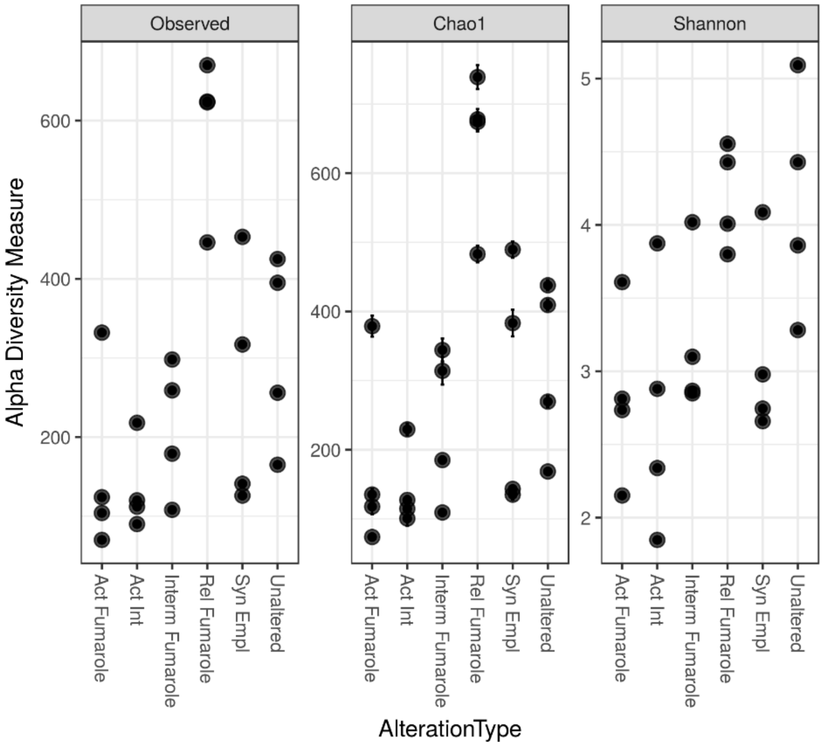

Figure S2. Rarefaction curves for the six Hawaii materials. The material type corresponding to each sample number can be found in Table S2.

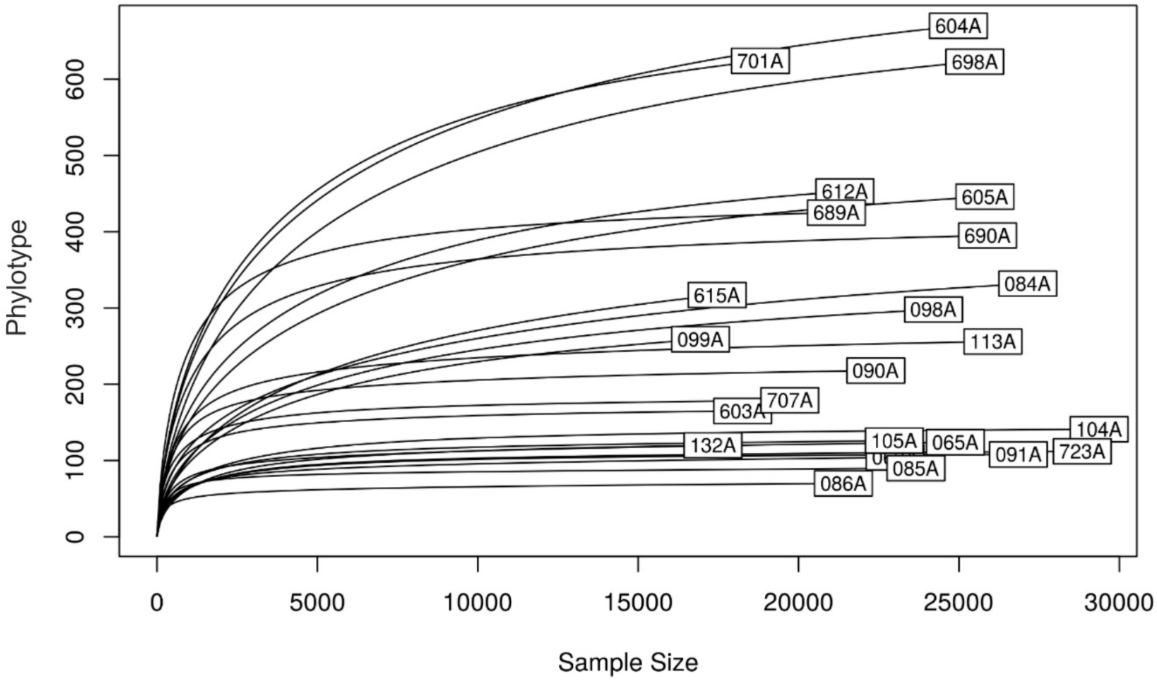

Supplement: Supplemental data [file Supp_Data.pdf]
